# The Practitioner's Encyclopædia of Medical Treatment

**Published:** 1915-12

**Authors:** 


**The Practitioner's Encyclopædia of Medical Treatmant.** Edited by W. LANGDON BROWN, M.D., F.R.C.P., and J. KEOGH MURPHY, M.C., F.R.C.S. With an Introduction by Sir THOMAS CLIFFORD ALLBUTT, K.C.B., M.D., F.R.S. Pp. xxiii. 874. London: Oxford Medical Publications. 1915.

Some ninety-six contributors have produced a compact single volume for the practitioner, up to date, and not of inordinate size or cost, written throughout by men of wide understanding and experience. The work is divided into two parts: (1) Methods of Treatment; (2) Agents in Treatment. Approximately one-third of the latter and two-thirds of the former, with a very voluminous but most useful index of over ninety pages in three columns. Details of surgical operative measures have not been included, although the indications for such measures and the general principles of the treatment of surgical cases could not well be excluded.

On looking through the long list of contributors, we notice the names of Dr. J. M. Fortescue-Brickdale, who writes on Coal-tar Derivatives, the Cinchona Alkaloids, Hypnotic and Narcotic Drugs and the Salicyl and Benzoyl Compounds, also that of Dr. J. A. Nixon, whose articles on Influenza and Pneumonia are quite useful guides, and Dr. A. Rendle Short, who describes the modern treatment of Tetanus.

The book as a whole appears to be a most reliable companion, to be consulted on all occasions when one desires to know the latest accepted views on any point of drug, dietetic, electro-therapeutic, X-ray, climatic, radium, ionic or serum treatment, and the masterly introduction by the Regius Professor of Physic at Cambridge should be read by all therapeutists.

